# The Taste Receptor TAS1R3 Regulates Small Intestinal Tuft Cell Homeostasis

**DOI:** 10.4049/immunohorizons.1900099

**Published:** 2020-01-24

**Authors:** Michael R. Howitt, Y. Grace Cao, Matthew B. Gologorsky, Jessica A. Li, Adam L. Haber, Moshe Biton, Jessica Lang, Monia Michaud, Aviv Regev, Wendy S. Garrett

**Affiliations:** * Department of Immunology and Infectious Diseases, Harvard T.H. Chan School of Public Health, Boston, MA 02115;; † Department of Molecular Metabolism, Harvard T.H. Chan School of Public Health, Boston, MA 02115;; ‡ Department of Pathology, Stanford University, Stanford, CA 94305;; § Broad Institute of MIT and Harvard, Cambridge, MA 02142;; ¶ Department of Biological Regulation, Weizmann Institute of Science, Rehovot 7610001, Israel;; ∥ Howard Hughes Medical Institute, Massachusetts Institute of Technology, Cambridge, MA 02142;; # Koch Institute for Integrative Cancer Research, Massachusetts Institute of Technology, Cambridge, MA 02142;; ** Department of Biology, Massachusetts Institute of Technology, Cambridge, MA 02142;; †† Department of Medical Oncology, Dana-Farber Cancer Institute, Boston, MA 02215

## Abstract

Tuft cells are an epithelial cell type critical for initiating type 2 immune responses to parasites and protozoa in the small intestine. To respond to these stimuli, intestinal tuft cells use taste chemosensory signaling pathways, but the role of taste receptors in type 2 immunity is poorly understood. In this study, we show that the taste receptor TAS1R3, which detects sweet and umami in the tongue, also regulates tuft cell responses in the distal small intestine. BALB/c mice, which have an inactive form of TAS1R3, as well as Tas1r3-deficient C57BL6/J mice both have severely impaired responses to tuft cell–inducing signals in the ileum, including the protozoa *Tritrichomonas muris* and succinate. In contrast, TAS1R3 is not required to mount an immune response to the helminth *Heligmosomoides polygyrus*, which infects the proximal small intestine. Examination of uninfected *Tas1r3*^*−*/−^ mice revealed a modest reduction in the number of tuft cells in the proximal small intestine but a severe decrease in the distal small intestine at homeostasis. Together, these results suggest that TAS1R3 influences intestinal immunity by shaping the epithelial cell landscape at steady-state. *ImmunoHorizons*, 2020, 4: 23–32.

## INTRODUCTION

Barrier defenses constitute the first arm of the innate immune system at mucosal surfaces. Via its sensing of mechanical injury, metabolites, or secreted molecules, the intestinal epithelium with its many cell subtypes is both an active defender and a signal broker to the innate and adaptive immune system. A chemosensory epithelial cell subset called tuft cells have recently emerged as critical sensors of intestinal helminths and protists and initiators of antiparasite immunity in the gut (1–3). Tuft cells produce IL-25, resulting in the accumulation of type 2 innate lymphoid cells (ILC2s) within the intestinal lamina propria and ILC2 production of IL-13 (1–3). This local increase in IL-13 biases the differentiation of epithelial progenitor/stem cells to become goblet cells, known for their production of mucins and antihelminthic resistin-like molecules, and tuft cells (1–4). The cross-talk between tuft cells and ILC2s creates a positive feedback loopamplifying the numbers of these cell types and culminating in productive antiparasite immunity.

Tuft cells use chemosensory signaling pathways to respond to parasites and initiate antiparasite immunity. Intestinal tuft cells express the canonical taste chemosensory components gustducin, 1-phosphatidylinositol 4,5-bisphosphate phosphodiesterase β−2 (PLCβ2), and transient receptor potential cation channel subfamily M member 5 (TRPM5) (1). InlingualtypeII taste cells,these chemosensory components facilitate signaling from G protein–coupled receptors (GPCRs) that transduce sweet, umami, or bitter tastes from food (5), whereas in the gut, they function to clear helminth infectionsand drive antiparasite immunity inresponse to *Tritrichomonas* protists (1). Small intestinal tuft cells also express the GPCR *Sucnr1* (*Gpr91*), which detects the metabolite succinate and initiates intestinal antiparasite immunity in a TRPM5-dependent manner (6–8). Mice lacking SUCNR1 do not respond to *Tritrichomonas* protists yet mount an effective immune response to the helminths *Heligomosomoides polygyrus* and *Nippostrongylus brasiliensis* (6, 8). In addition, bitter-taste GPCRs were recently implicated in the tuft cell response to infection with the helminth *Trichinella spiralis*(9). Collectively, these observations suggest that tuft cells use multiple taste-associated GPCRs to initiate intestinal antiparasite immunity. Given the immense diversity of microbial and nonmicrobial signals that stimulate the antiparasite immune response at barrier surfaces, we hypothesized that there may be additional uncharacterized tuft cell receptors that modulate intestinal antiparasite immunity.

In this study, we show that the taste receptor, TAS1R3, which mediates responsiveness to sweet and umami in the tongue, also contributes to antiparasite immunity in the distal small intestine. Stimuli that induce a tuft cell response in the ileum, such as *Tritrichomonas muris* or succinate, are severely diminished in Tas1r3^−/−^ mice. Our results reveal important roles for TAS1R3 in regulating tuft cell homeostasis in the small intestine, thereby modulating sensitivity to luminal stimuli during the initiation of type 2 immunity.

## MATERIALS AND METHODS

### Mice

Wild-type (WT) C57BL/6J mice were bred and housed in microisolator cages in the specific-pathogen-free (SPF) barrier facility at the Harvard T.H. Chan School of Public Health and at Stanford University. BALB/c, Gfi1b^eGFP/+^, Plcb2^−/−^, and Stat6^−/−^ mice were obtained from The Jackson Laboratory (Bar Harbor, ME). C57BL/6J Tas1r3^−/−^ and *Trpm5*^*−*/−^ mice (10, 11) were generously provided by Dr. R. Margolskee (Monell Chemical Senses Center), and *Gpr91*^*−*/−^ mice were generously provided by Dr. J. Peti-Peterdi with permission from Amgen (12). All genetically modified mouse lines were bred on a C57BL6/J background and were cohoused for at least 2 wk prior to the experiments. *Gfi1b*^*eGFP*/+^ *Gpr91*^−/−^, *Gfi1b*^*eGFP*/+^ *Plcb2*^−/−^, *Gfi1b*^*eGFP*/+^ *Stat6*^−/−^, *Gfi1b*^*eGFP*/+^ *Tas1r3*^−/−^, and *Gfi1b*^*eGFP*/+^ *Trpm5*^−/−^ mice were generated by breeding *Gfi1b*^*eGAQFP*/+^ mice to the relevant knockout mice. Germ-free (GF) WT C57BL/6J mice were bred and maintained in semirigid gnotobiotic isolators in the Harvard T. H. Chan Gnotobiotic Center for Mechanistic Microbiome Studies. Mice were used experimentally between 6 and 12 wk of age. Animal studies and experiments were approved and carried out in accordance with Harvard Medical School’s Standing Committee on Animals, Stanford’s Institutional Animal Care and Use Committee, and the National Institutes of Health guidelines for animal use and care.

### Infection with protozoa and helminths

*T*. *muris* was isolated and cultured from bred-in-house mice as described previously (1). A total of 5 × 10^6^ T. *muris* were orally administered to mice. Mice were then sacrificed 16–18 d postinfection.

*H. polygyrus* maintenance and infection was performed as previously described (13). Mice were orally infected with 200 L3 larvae and sacrificed 6 wk later. The proximal 15 cm of small intestine was excised, and worms were counted with a dissection microscope.

### T. muris enumeration

*T. muris* load in the distal small intestine was enumerated as previously described (1). The distal 10 cm of small intestine was removed and flushed with ice-cold sterile PBS using a 19-gauge feeding needle. The intestinal contents were then pelleted by centrifugation and stored at −20°C. Genomic DNA was isolated from the stool with QIamp Fast DNA Stool Mini Kit (Qiagen) according to the manufacturer’s directions. To detect and enumerate *T. muris*, quantitative PCR (qPCR) was performed using KAPA SYBR Fast Universal PCR (KAPA Biosystems) with the following primers recognizing the *T. muris* 28S rRNA gene: 59-GCTTTTGCAAGCTAGGTCCC-39and 59-TTTCTGATGGGGCG TACCAC-39. These qPCR values were converted to *T. muris* numbers using a standard curve generated using known amounts of *T. muris*.

### Metabolite/taste receptor ligand feeding

For succinate treatment, mice were provided with 100 mM succinic acid disodium salt (no. 224731; Sigma-Aldrich) in the drinking water for 1, 2, or 4 wk as indicated. Two hundred millimolar sodium chloride was used as a control to match sodium molarity. Sucralose was fed at concentrations of 2, 20, or 100 mM in the drinking water, and rebaudioside A was fed at a concentration of 1 mM for 8 d.

### IL-25 injection

Mice were i.p. injected daily with 0.5 μg of rIL-25 (endotoxin level, 1.0 endotoxin unit per 1 μg; R&D Systems) or equivalent volume of sterile PBS for 7 d before harvesting the distal small intestine.

### Epithelial cell isolation and flow cytometry

The small intestine and/or colon were removed and flushed with ice-cold sterile PBS using a 19-gauge feeding needle. The small intestine was further subdivided into the duodenum (proximal 7 cm), jejunum (next 10 cm), and ileum (distal 10 cm), and Peyer patches were removed. Both small intestinal regions and colons were then opened longitudinally and gently agitated at 4°C in PBS, 2% FBS, 5 mM HEPES, and 1 mM DTT for 10 min. The tissue was then transferred into prewarmed PBS, 2% FBS, 5 mM HEPES, and 5 mM EDTA and rotated at 37°C for 15 min followed by vigorous shaking to remove epithelial cells. This was repeated and epithelial cells from both fractions were combined and washed with PBS. The epithelium was then digested in DMEM containing 10% FBS, 0.5 U/ml Dispase II (StemCell Technologies), and 50 μg/ml DNase (Roche) for 10 min at 37°C. The resulting solution was passed through 40-μm filters and washed with PBS, 2% FBS, and 1 mM EDTA. The resulting single-cell suspension was initially Fc blocked with anti-CD16/CD32 (clone 93; BioLegend) and then stained with the following Abs: PacBlue-conjugated anti-CD45 (clone 30-F11; BioLegend) and allophycocyanin-conjugated anti-EpCam (clone G8.8; BioLegend). Cell viability was assessed by propidium iodide (BioLegend) staining.

### Histology and fluorescence microscopy

The small intestine was removed and divided into proximal and distal sections before overnight fixation in 4% paraformaldehyde. The tissue was then embedded in paraffin and cut into 5-μm thick sections. For both histology and immunofluorescence, sections were initially deparaffinized and rehydrated. Goblet cells were identified by Alcian blue/nuclear red staining and enumerated along the crypt-villus axis by quantitative microscopy. For immunofluorescence, heat-mediated Ag retrieval was performed in Tris-EDTA buffer 0.05% Tween-20 (pH 9) for 20 min. Afterwards, the slides were washed in PBS and blocked in PBS containing 3% BSA, 3% donkey serum, 0.1% Triton X-100, and 0.1% saponin for 1 h at room temperature. Primary Abs were incubated overnight at 4°C and secondary Abs were applied for 1.5 h at room temperature. Primary Abs included the following: rabbit anti-DCLK1 (1:250 dilution, ab37994; Abcam) and mouse anti–E-cadherin (1:400 dilution, 36/E-cadherin; BD Biosciences). DNA was labeled with DAPI (0.5 μg/ml).

### RNA isolation and RT-PCR

Epithelial cells from *Gfi1b*^*eGFP/+*^ mice were isolated and stained for FACS as previously detailed. Tuft cells were sorted as GFP^+^EpCam^+^CD45^−^PI^−^, whereas the remaining epithelial cells were GFP^−^EpCam^+^CD45^−^PI^−^. RNA was then extracted fromtuft cells and the remaining epithelium using RNeasy Micro Kit (Qiagen). For RNA isolation from total epithelium, the epithelial fraction was collected after the EDTA wash (as described above) and lysed in Qiazol (Qiagen) for RNA extraction following manufacturer’s instructions. cDNA was synthesized using the iScript cDNA Synthesis Kit (Bio-Rad), and quantitative RT-PCR was performed using the PowerUp SYBR Green Master Mix qPCR Kit (Applied Biosystems). The following primers were used: Dclk1, 5′-CAGCCTGGACGAGCTGGTGG-3′, 5′-TGACCAGTTGGGGTTCACAT-3′; Trpm5, 5′-CCTCCGT GCTTTTTGAACTCC-3′, 5′-CATAGCCAAAGG TCGTTCCTC-3′; Klf4, 5′-ATCCTTTCCAACTCGCTAACCC-3′, 5′-CGGATCGGATA GCTGAAGCTG-3′; Muc2, 5′-CCATTGAGTTTGGGAACATGC-3′, 5′-TTCGGCTCGGTGTTCAGAG-3′; Chga, 5′-CAGGCTACAAAG CGATCCAG-3′, 5′-GCCTCTGTCTTTCCATCTCC-3′; Tph1, 5′-AACAAAGACCATTCCTCCGAAAG-3′, 5′-TGTAACAGG CTCACATGATTCTC-3′; Def20, 5′-TGTAGAAAAGGAGGC TGCAATAG-3′, 5′-AGAACAAAAGTCGTCCTGAGC-3′; Lyz1, 5′GCCAAGGTCTACAATCGTTGTGAGTTG-3′, 5′-CAGTCA GCCAGCTTGACACCACG-3′; Lgr5, 5′-CCTACTCGAAGACT TACCCAGT-3′, 5′-GCATTGGGGTGAATGATAGCA-3′; Ascl2, 5′-GCCTACTCGTCGGAGGAA-3′, 5′-CCAACTGGAAAA GTCAAGCA-3′; Gapdh, 5′-CCTCGTCCCGTAGACAAAATG-3′, 5′-TCTCCACTTTGCCACTGCAA-3′;Tas1r3, 5′-AGGTGGCT CACAGTTCTGCT-3′, 5′-GAGGTGAGCCATTGGTTGTT-3′.

Data are presented as relative expression normalized to Gapdh.

### Small intestine organoid culture and flow cytometry

Distal small intestinal organoids were prepared as previously described (14). When indicated, IL-13 (10 ng/ml, endotoxin level < 0.01 ng/μg; BioLegend) was added to organoid media for 48 h. To perform flow cytometry, organoids were liberated from the matrigel matrix as described (14) and digested in DMEM containing 10% FBS, 0.5 U/ml Dispase II (StemCell Technologies), and 50 μg/ml DNase (Roche) for 8 min at 37°C. The resulting solution was filtered through 40-μm mesh and stained for flow cytometry with allophycocyanin-conjugated anti-EpCam (clone G8.8; BioLegend) with cell viability assessed with propidium iodide (BioLegend).

### Statistics

The Mann–Whitney *U* test was used to compare two samples. One-way ANOVA was used for multiple-group comparisons followed by Holm–Sidak post hoc testing. The *p* values are indicated in the figure legends.

### RNA sequencing

#### Experimental procedure.

Epithelial cells from *Gfi1b*^*eGFP*/+^ mice were isolated and stained for FACS as previously detailed. Tuft cells were sorted as GFP^+^EpCam^+^CD45^−^PI^−^ directly into lysis buffer from the RNeasy Mini Kit (Qiagen). RNA was isolated according to the manufacturer’s protocol and RNA integrity was confirmed using a 2100 Agilent BioAnalyzer.

#### Library preparation.

Libraries were prepared using a modified SMART-Seq2 protocol as previously reported (15). RNA lysate cleanup was performed using RNAClean XP beads (Agencourt), followed by reverse transcription with Maxima Reverse Transcriptase (Life Technologies) and whole transcription amplification (WTA) with KAPA HotStart HIFI 2× ReadyMix (Kapa Biosystems) for 18 cycles. WTA products were purified with Ampure XP beads (Beckman Coulter), quantified with Qubit dsDNA HS Assay Kit (Thermo Fisher Scientific), and assessed with a high sensitivity DNA chip (Agilent). RNA-sequencing libraries were constructed from purified WTA products using Nextera XT DNA Library Preparation Kit (Illumina). The libraries were sequenced on an Illumina NextSeq 500.

#### Computational analysis.

BAM files were converted to merged, de-multiplexed FASTQs using the Illumina-provided Bcl2Fastq software package v2.17.1.14. Paired-end reads were mapped to the UCSC mm10 mouse transcriptome using Bowtie (16) with parameters “-q–phred33-quals -n 1 -e 99999999 -l 25 -I 1 -X 2000 -a -m 15 -S -p 6,” which allows alignment of sequences with one mismatch. Expression levels of genes were quantified as transcript-per-million values calculated by RSEM (17) v1.2.3 in paired-end mode. Negativebinomial models werefittedto thedata and likelihood-ratio tests used to assess differential expression, implemented using the DESeq2 package in R (18).

All data are deposited in GEO (GSE140395), https://www.ncbi.nlm.nih.gov/geo/query/acc.cgi?acc=GSE140395.

## RESULTS

### C57BL6/J and BALB/c mice differ in tuft cell response to the protozoa T. muris but not the helminth H. polygyrus

**FIGURE 1.**
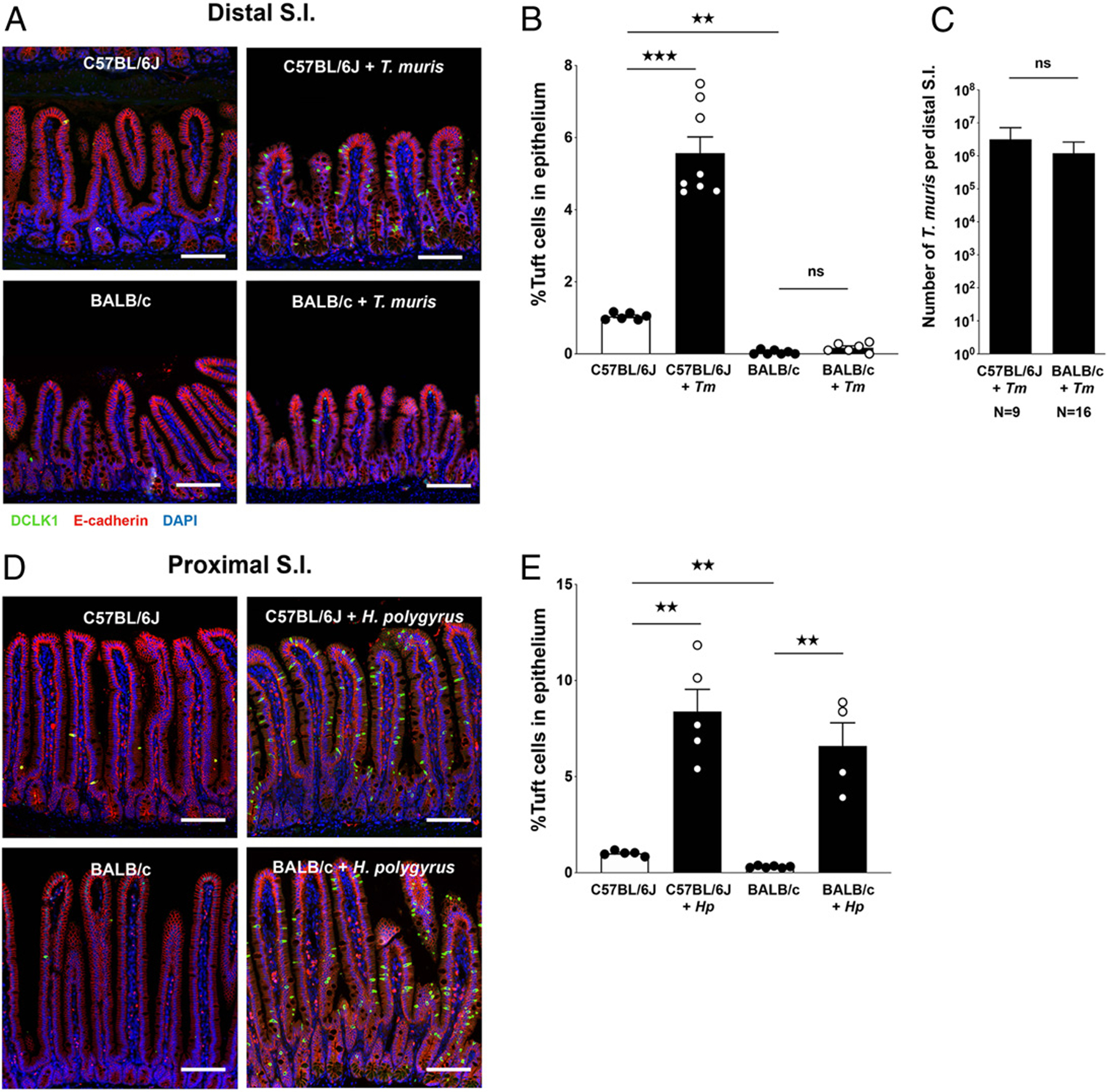
Tuft cell responses differ in C57BL/6J and BALB/c mice to the protozoa *T. muris* but not the helminth *H. polygyrus*. (**A**) Representative distal small intestine (SI) images from uninfected and *T. muris*–colonized C57BL/6J and BALB/c mice (scale bar, 100 μm) and (**B**) tuft cell frequency at day 18 postinfection. (**C**) *T. muris* abundance in distal SI contents determined by qPCR. (**D**) Representative proximal SI images from uninfected and *H. polygyrus*–infected C57BL/6J and BALB/c mice (scale bar, 100 mm) and (**E**) tuft cell frequency at day 14 postinfection. Each symbol represents an individual mouse, and all data are representative of two independent experiments. Data are plotted as means with SEM. ***p < 0.001, **p < 0.01. n.s., not significant using the Mann–Whitney *U* test.

Given the critical role of chemosensation in tuft cell–mediated antiparasite immunity in the gut, we considered if inbred mouse strains with their well-characterized genetics and immune responses to parasites could be used to further elucidate the potential connection between parasite surveillance and taste receptors. Inbred mouse strains such as BALB/c and C57BL/6J can differ in their immune responses to parasites, which results in marked differences in clearing these infections (19, 20). Furthermore, BALB/c and C57BL/6J mice carry genetic polymorphisms in the taste receptor Tas1r3, with C57BL/6J having a high preference for the TAS1R3 ligand saccharin and BALB/c having a low preference (21, 22). To investigate if these differences between mouse strains could alter the tuft cell response to parasites, we colonized both C57BL/6J and BALB/c mice with the protozoa *T. muris. T. muris* induced a significant increasein tuft cell frequency at the site of colonization, the distal small intestine (ileum) (1), of C57BL6/J mice, which contrasted with an absence of tuft cell expansion in *T. muris*–colonized BALB/c mice (Fig. 1A, 1B). This difference in tuft cell responses was not due to variation in *T. muris* colonization, as protozoal loads were similar between C57BL/6J and BALB/c mice (Fig. 1C).

Intestinal helminth–induced immunity is also accompanied by a significant increase in tuft cell frequency (1–3). Consistent with these prior results, BALB/c and C57BL/6J mice both showed robust expansion of tuftcellsinthe proximal small intestineduring *H. polygyrus* infection (Fig. 1D, 1E), confirming that BALB/c mice can generate tuft cells in response to some parasitic stimuli. To determine if the defect in response to *T. muris* was specific to the protozoa or a more general feature of the ileum, we injected BALB/c mice with rIL-25. This cytokine bypasses microbial signal–dependent pathways to directly expand tuft cells via induction of IL-13 production by ILC2s (1–3). IL-25 injection resulted in tuft cell expansion in the distal small intestine (Supplemental Fig. 1), suggesting that the lack of BALB/c response to *T. muris* was not due to an inability to increase tuft cells in the ileum.

### Tuft cells express the type 1 taste receptor TAS1R3, which affects their response to expansion stimuli

**FIGURE 2.**
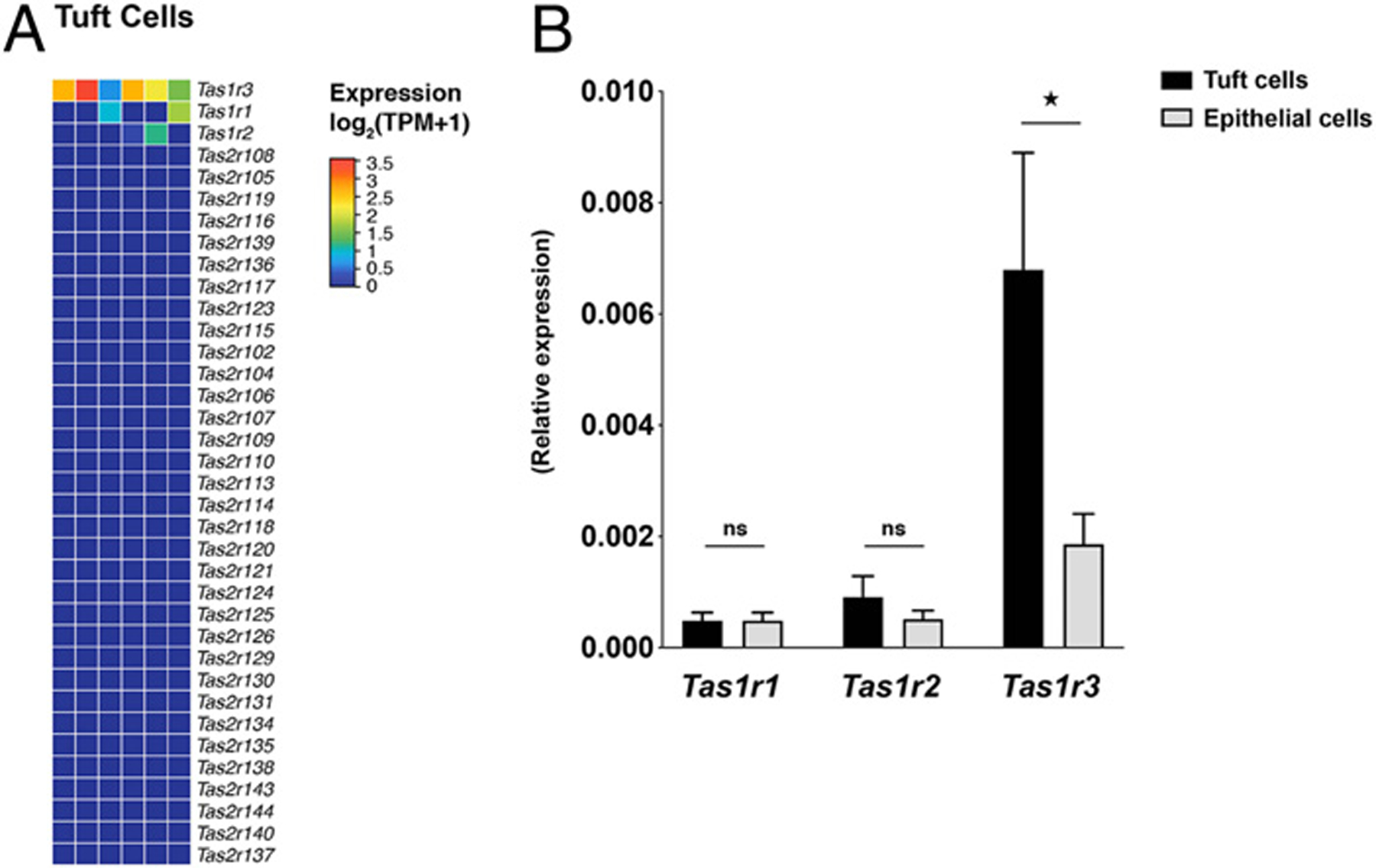
Small intestinal tuft cells express the type 1 taste receptor *Tas1r3*. (**A**) Type 1 and 2 taste receptor expression in sorted C57BL/6J distal small intestinal tuft cells determined by RNA-sequencing. (**B**) Type 1 taste receptor gene expression in sorted distal small intestine tuft cells determined by qPCR compared with the non–tuft cell epithelium. Data represent six independent samples (A) or two independent experiments (B). Data are plotted as means with SEM. *p < 0.05, Mann-Whitney *U* test.

To understand the mechanism underlying the lack of tuft cell response to *T. muris* in BALB/c mice, we returned to the observation that polymorphisms in *Tas1r3* severely reduce the sensitivity of this receptor in BALB/c mice (21, 22). In lingual taste, TAS1R3 activates a signal transduction pathway involving PLCb2 and TRPM5, which are chemosensory components used by tuft cells to respond to *T. muris* (1). Previous reports found that tuft cells in the stomach, trachea, thymus, gallbladder, and urethra express Tas1r3 (6, 23, 24), but the level of *Tas1r3* expression by intestinal tuft cells was unclear. We sorted ileal tuft cells from C57BL/6J mice and used RNA sequencing to measure the expression of type 1 and type 2 taste receptors, which signal through the transduction components PLCβ2 and TRPM5. *Tas1r3* had the highest expression, whereas most other receptor genes were not detected (Fig. 2A). Quantitative RT-PCR analysis confirmed this result (Fig. 2B), consistent with single-cell sequencing analysis of small intestinal epithelial cells that identified *Tas1r3* as a tuft cell marker (25).

**FIGURE 3.**
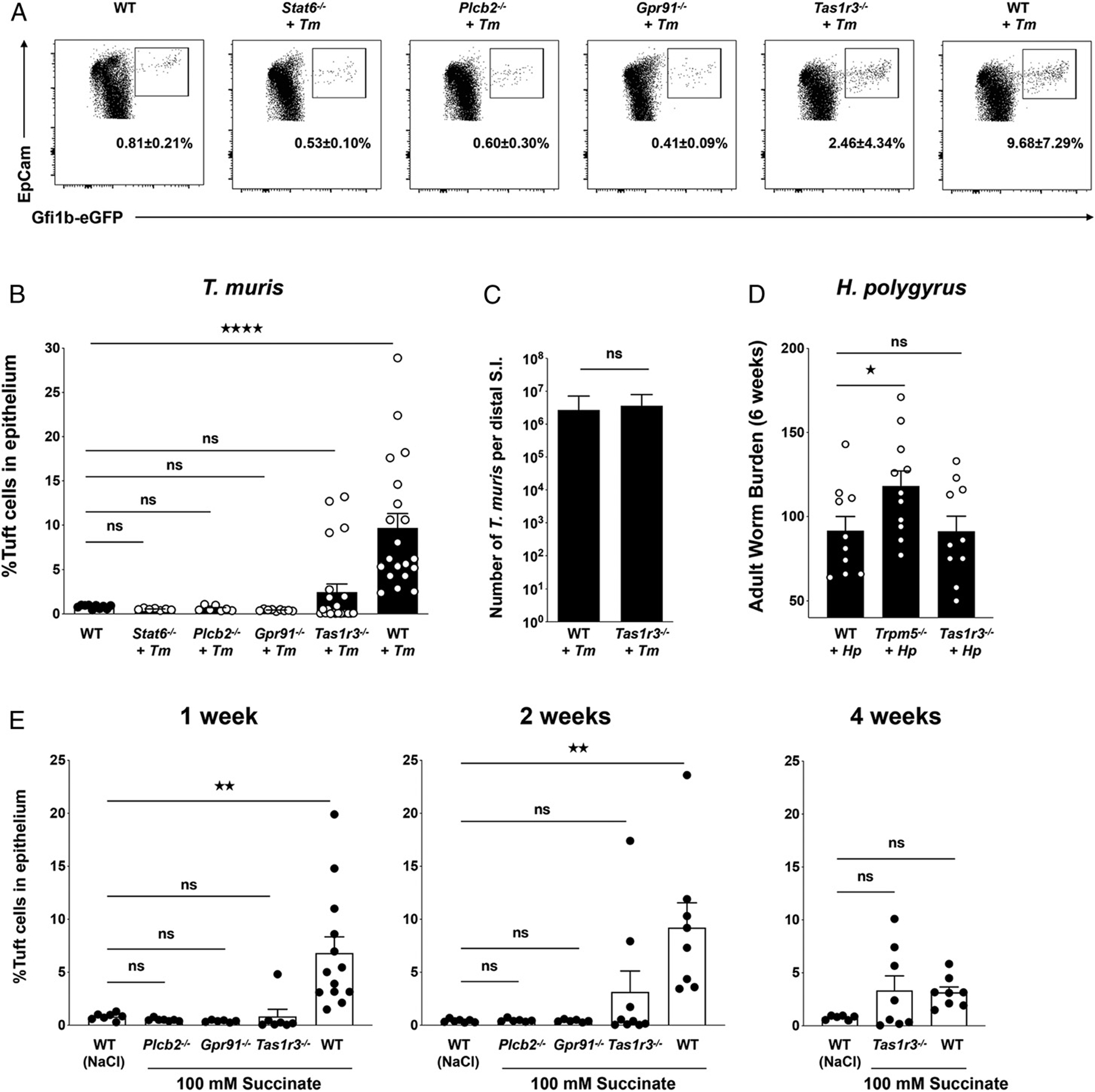
TAS1R3 affects distal small intestinal tuft cell response to expansion stimuli. (**A**) Representative flow cytometry plots of live, CD45^−^EpCAM^+^ intestinal epithelial cells from uninfected or *T. muris*–colonized WT (*Gfi1b*^*eGFP/+*^) C57BL/6J, *Gfi1b*^*eGFP/+*^ *Stat6*^−/−^, *Gfi1b*^*eGFP*/+^ *Plcb2*^−/−^, *Gfi1b*^*eGFP/+*^ *Gpr91*^−/−^, and *Gfi1b*^*eGFP/+*^ *Tas1r3*^−/−^ mice and (B) tuft cell frequency. (**C**) *T. muris* abundance in distal small intestine (SI) contents determined by qPCR. (**D**) *H. polygyrus* worm burden in proximal SI at 6 wk postinfection. (**E**) Tuft cell frequency determined by flow cytometry after 1, 2, or 4 wk of feeding succinate or NaCl in the drinking water. Each symbol represents an individual mouse, and all data are representative of at least two independent experiments. Data are plotted as means with SEM. ****p < 0.0001, **p, 0.01, *p < 0.05, one-way ANOVA with Holm–Sidak test or Mann–Whitney *U* test. ns, not significant.

We hypothesized that if the inactive BALB/c TAS1R3 contributed to impaired BALB/c responses to *T. muris*, then Tas1r3^−/−^ C57BL/6J mice should recapitulate this lack of response. Indeed, tuft cell frequency in Tas1r3^−/−^ mice did not significantly increase after *T. muris* colonization (Fig. 3A, 3B). Similar to what we observed in BALB/c mice, this defect was not due to lower *T. muris* colonization (Fig. 3C). Furthermore, the effect of TAS1R3 was restricted to *T. muris*, as *Tas1r3*^−/−^ mice had a similar ability to expel *H. polygyrus* as WT mice, whereas *Trpm5*^−/−^ mice had an increased worm burden as previously described (Fig. 3D) (1).

Although tuft cell frequency remained unchanged in the majority of *Tas1r3*^−/−^ mice after *T. muris* colonization, we noted that a subset of mice exhibited a strong tuft cell response. This bimodal response was not observed in mice lacking other tuft cell–regulating components, including PLCβ2, STAT6, (a transcription factor downstream of IL-13R signaling), and GPR91 (a succinate receptor reported to be involved in sensing of *Tritrichomonas* species) (6, 7) (Fig. 3A).These observations led us to question whether TAS1R3 regulates tuft cell expansion through sensing of a *T. muris*–associated signal, such as succinate, or potentially another mechanism.

Feeding mice succinate in the drinking water induces a significant expansion of tuft cells in the distal small intestine, which highly resembles the tuft cell response to *Tritrichomonas* colonization (6–8). WT mice fed 100 mM succinate in the drinking water for 1 wk exhibited tuft cell hyperplasia (Fig. 3E). However, similar to *Gpr91*^−/−^ and *Plcb2*^−/−^mice, feeding succinate to *Tas1r3*^−/−^ mice for 1 wk failed to increase tuft cell levels in the distal small intestine (Fig. 3E). To assess the kinetics of tuft cell response tosuccinate, we also fed mice 100 mM succinate for 2 and 4 wk. In contrast to 1 wk of succinate feeding, these longer succinate exposures increased the proportion of *Tas1r3*^−/−^ mice with enhanced tuft cell abundance. These findings suggest that TAS1R3 is not required to sense succinate but may act to increase the response kinetics of tuft cells to expansion stimuli.

### TAS1R3 regulates homeostatic tuft cell abundance

**FIGURE 4.**
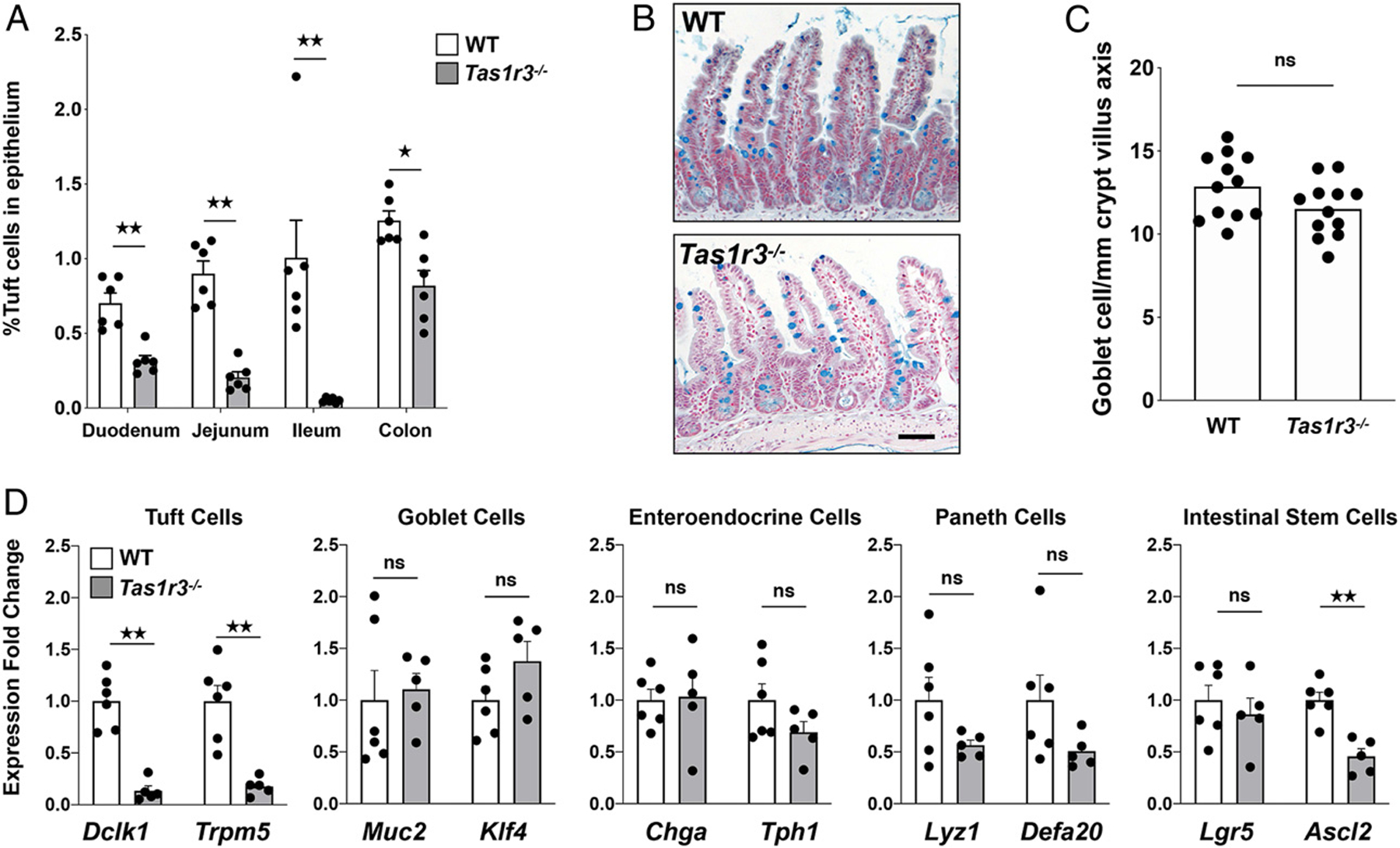
TAS1R3 regulates homeostatic tuft cell abundance. (**A**) Tuft cell frequency determined by flow cytometry in the duodenum, jejunum, ileum, and colon of C57BL/6J WT (*Gfi1b*^*eGFP/+*^) and *Tas1r3*^−/−^ (*Gfi1b*^*eGFP/+*^ *Tas1r3*^−/−^) at steady-state. (**B**) Representative distal small intestine images of Alcian blue and nuclear red staining (scale bar, 50 μm) and (**C**) goblet cell frequency at steady-state. (**D**) Expression of epithelial cell subset marker genes determined by qPCR in the epithelial fraction of the distal small intestine of WT and *Tas1r3*^−/−^ mice at steady-state. Each symbol represents an individual mouse, and all data are representative of three independent experiments. Data are plotted as means with SEM. **p, 0.01, *p, 0.05, one-way ANOVA with Holm–Sidak test or Mann–Whitney *U* test. ns, not significant.

Tuft cells function in a feed-forward loop in which tuft cell–derived IL-25 leads to increased IL-13 production by ILC2s and expansion of Th2 cells, which results in additional tuft cells and further production of IL-25. Therefore, we hypothesized that the response kinetics of tuft cells to a stimulus would be affected by the number of tuft cells that are available to initiate the feed-forward loop. To determine if TAS1R3 influences tuft cell frequency at steady-state, we profiled tuft cell populations across different regions of the gut and found that *Tas1r3*^−/−^ mice had fewer tuft cells compared with WT, with the most pronounced defect in the ileum (Fig. 4A). We further verified the relative reduction of tuft cells in the ileal epithelium of *Tas1r3*^−/−^ animals by measuring RNA expression of the tuft cell markers *Dclk1* and *Trpm5* and by immunofluorescence microscopy, which revealed markedly fewer DCLK1^+^ tuft cells in the absence of TAS1R3 (Fig. 4D, Supplemental Fig. 2). Because IL-13 stimulates epithelial progenitor cells to differentiate into both tuft cells and goblet cells (1–4), we also examined the number of goblet cells in the ileum of WT and *Tas1r3*^−/−^ mice at steady-state but found no significant difference (Fig. 4B, 4C). We confirmed that other epithelial populations remained largely unchanged in *Tas1r3*^−/−^ mice by measuring the expression of representative gene markers for intestinal epithelial cell subsets (25) (Fig. 4D). One notable exception was the stem cell–regulating gene *Ascl2*. Although *Ascl2* is a signature gene associated with intestinal epithelial stem cells, it is also expressed by tuft cells (26) and therefore the decrease in *Ascl2* expression in the epithelium of *Tas1r3*^−/−^ mice may reflect the reduced number of tuft cells rather than changes in the stem cell population. Therefore, we surmised that TAS1R3 is required for maintenance of homeostatic levels of tuft cells and that the few ileal tuft cells present in *Tas1r3*^−/−^ mice cannot trigger the IL-25–and IL-13–dependent feedback loop required for expansion with the same kinetics seen in WT mice.

**FIGURE 5.**
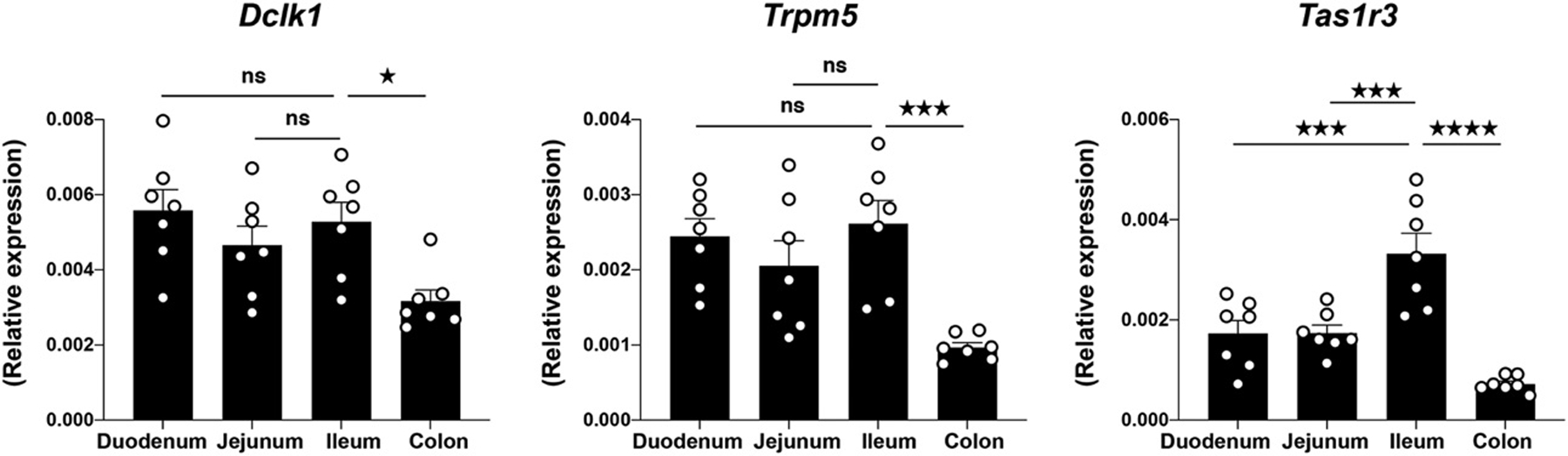
Epithelial expression of TAS1R3 is highest in the ileum. Expression of *Dclk1*, *Trpm5*, and *Tas1r3* determined by qPCR in the epithelial fraction of the indicated intestinal region in WT C57BL/6J mice. Data are plotted as means with SEM. ****p, 0.0001, ***p, 0.001, *p, 0.05, one-way ANOVA with Holm–Sidak test. ns, not significant.

**FIGURE 6.**
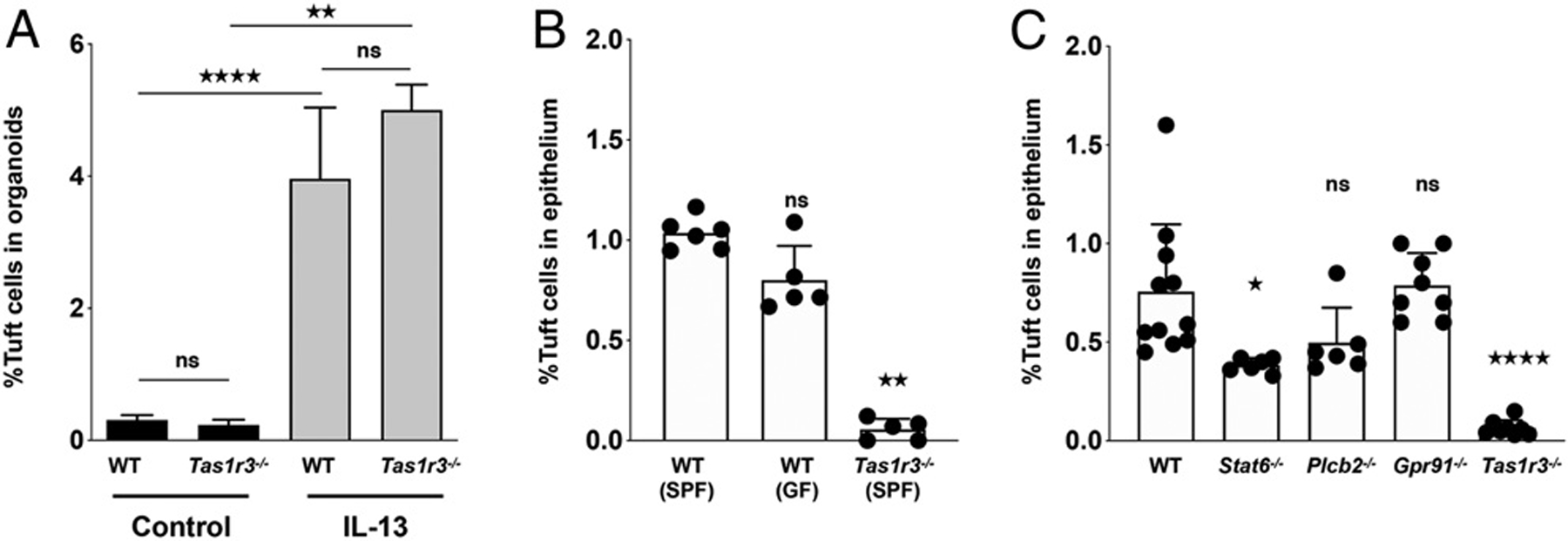
Analysis of potential TAS1R3 ligands and signaling pathways. (**A**) GFP^+^ tuft cell abundance determined by flow cytometry of WT and *Tas1r3*^−/−^ distal small intestine (SI) organoids with or without 48-h rIL-13 treatment. (**B**) Distal SI tuft cell abundance determined by immunofluorescence in SPF and GF mice. (**C**) Distal SI tuft cell abundance determined by flow cytometry at steady-state. Each symbol represents an individual mouse, and all data are pooled from at least two independent experiments. Data are plotted as means with SEM. ****p, 0.0001, **p, 0.01, *p, 0.05, Mann–Whitney *U* test. ns, not significant.

To understand how TAS1R3 regulates intestinal tuft cells, we next measured the expression of *Tas1r3*, *Dclk1*, and *Trpm5* in epithelial cells harvested from the duodenum, jejunum, ileum, and colon of WT mice. Although the expression of the tuft cell markers *Dclk1* and *Trpm5* was relatively consistent across the small intestine, Tas1r3 expression was elevated in the ileum (Fig. 5). This pattern of *Tas1r3* expression inversely correlated with the severity of the tuft cell defect in *Tas1r3*^−/−^ mice. To identify intestinal TAS1R3 ligands that alter tuft cell frequency, we generated organoids from the ileal epithelium of *Gfi1b*^*eGFP/+*^ and *Gfi1b*^*eGFP/*+^ × *Tas1r3*^−/−^ mice. In this system, TAS1R3 did not affect baseline tuft cell abundance or the IL-13–induced increase in tuft cell frequency (Fig. 6A). This observation led us to consider potential sources of TAS1R3 ligands in vivo. The microbiota is a rich source of ligands for host receptors, so we compared the number of tuft cells in the ileum of conventionally raised SPF WT and *Tas1r3*^−/−^ mice with GF mice. GF mice had a similar frequency of tuft cells as conventional WT mice but significantly more tuft cells than *Tas1r3*^−/−^ mice (Fig. 6B), suggesting that the microbiota is not an important source of TAS1R3 ligands in the ileum.

In lingual taste cells, TAS1R3 forms a heterodimer either with TAS1R1 to detect umami flavor or TAS1R2 to detect sweet flavor (27). However, in the ileum, we observed considerably lower expression of *Tas1r1* and *Tas1r2* compared with *Tas1r3* (Fig. 2A), suggesting that many common sweet- and umami-taste agonists may not stimulate intestinaltuft cells. Therefore, tomimic adietary agonist of TAS1R3, we fed mice the artificial sweetener sucralose, which has been reported to target TAS1R3 directly (28). After feeding mice 2, 20, or 100 mM sucralose in the drinking water for 8 d, we did not observe a significant change in tuft cell abundance in either the duodenum or the ileum (Supplemental Fig. 3). Similarly, rebaudioside A, a steviol glycoside that potentiates TRPM5 signaling (29), did not increase tuft cell frequency (Supplemental Fig. 3). Because the TAS1R3 receptor putatively activates the canonicaltastesignal transductionpathway involving PLCβ2, we measured the frequency of tuft cells in the ileum of *Plcb2*^−/−^ mice at homeostasis. Surprisingly, there were significantly fewer tuft cells in *Tas1r3*^−/−^ mice than *Plcb2*^−/−^ mice (Fig. 6C). This defect was not observed in mice lacking another tuft cell–regulating GPCR, GPR91. Additionally, there were fewer tuft cells in *Tas1r3*^−/−^ than in *Stat6*^−/−^ mice (Fig. 6C). Together, these findings imply that TAS1R3 may not regulate tuft cell abundance through currently established taste chemosensory pathways or through IL-13/IL-4 activation of STAT6 signaling.

## DISCUSSION

In this study, we found that the canonical taste receptor TAS1R3 regulates homeostatic tuft cell abundance, particularly in the ileum, where the frequency of tuft cells is reduced by ~95% in *Tas1r3*^−/−^ mice. The loss of tuft cells significantly decreases the sensitivity of *Tas1r3*^−/−^mice to antiparasite immunity–inducing stimuli like *T. muris* and succinate. In contrast to previous work on tuft cell receptors, which focused on the direct sensing of parasite products (6, 7, 9), our study revealed that TAS1R3 indirectly affects intestinal immunity by shaping epithelial cell subset composition at baseline. The bimodal distribution of high and low tuft cell levels in *Tas1r3*^−/−^ mice fed succinate, with an increasing proportion of mice with high levels of tuft cells after longer treatment, suggests that the lower baseline levels of tuft cells in *Tas1r3*^−/−^ mice delay the feed-forward expansion loop. Although the tuft cell response to succinate in *Tas1r3*^−/−^ mice can be partially compensated for over time, even after 4 wk of succinate treatment, ~50% of *Tas1r3*^−/−^ mice display no change in tuft cell abundance. This observation is consistent with several possibilities. The relatively low frequency of tuft cells at baseline in *Tas1r3*^−/−^ mice may slow the response kinetics in some animals, whereas others may never reach the threshold necessary to engage the tuft cell feed-forward loop. Alternatively, the kinetics may suggest a role for other cell populations expressing TAS1R3.

We confirmed that tuft cells in the ileum express *Tas1r3*, but we cannot exclude the influence of *Tas1r3* expression by other cell types on tuft cell frequency. For example, a subtype of enteroendocrine cell, the L cell, also expresses *Tas1r3* (30). L cells are particularlyabundant in the distal small intestine and colon and are characterized in part by the secretion of the hormones GLP-1 and GLP-2. These peptides function as incretin hormones but can also remodel the gut through increased small intestinal growth and epithelial proliferation (31, 32). GLP-1 secretion by duodenal L cells is decreased in *Tas1r3*^−/−^ mice (33), but the effect of these peptides on the differentiation or stability of tuft cells is unknown. Nonetheless, our data support that among the epithelial subsets, tuft cells are uniquely reduced in *Tas1r3*^−/−^ mice. However, intestinal tuft cells are heterogeneous (25, 34), and future work is needed to determine if TAS1R3 supports the differentiation or stability of all tuft cells or only specific tuft cell subpopulations.

To identify endogenous ligands for TAS1R3, we investigated the role of both the gut microbiota and specific dietary sugars on tuft cell frequency. The ilea of GF and conventionally raised SPF mice contained a similar abundance of tuft cells and significantly more tuft cells than conventionally raised SPF *Tas1r3*^−/−^ mice. This observation suggests that microbial stimuli do not stimulate TAS1R3 to sustain normal tuft cellfrequency. Mice fedthe artificial sweetener sucralose also failed to expand tuft cells in either the duodenum or the ileum. Future efforts to identify intestinal TAS1R3 ligands may be aided by determining whether intestinal TAS1R3 functions as a homodimer or a heterodimer with another class C GPCR. Additionally, our study highlights a potential opportunity to understand intestinal tuftcell regulationin humans. TAS1R3 is expressed within the human intestine (35, 36), and natural genetic variation in *TAS1R3* accounts for significant differences in sweet perception among the population (37). Whether genetic differences in TAS1R3 can regulate human intestinal tuft abundance and type 2 immunity awaits further investigation.

## Supplementary Material

1

## Abbreviations used in this article:

GF: germ-free
GPCR: G protein–coupled receptor
ILC2: type 2 innate lymphoid cell
qPCR: quantitative PCR
SPF: specific-pathogen-free
TRPM5: transient receptor potential cation channel subfamily M member 5
WT: wild-type
WTA: whole transcription amplification
