# Supplementary material for: The Taste Receptor TAS1R3 Regulates Small Intestinal Tuft Cell Homeostasis
Source: Immunohorizons. Author manuscript; Available in PMC 2020 May 4. (PMC7197368; doi:10.4049/immunohorizons.1900099)
Supplement: 1 [file NIHMS1582159-supplement-1.pdf]

Fig. S1

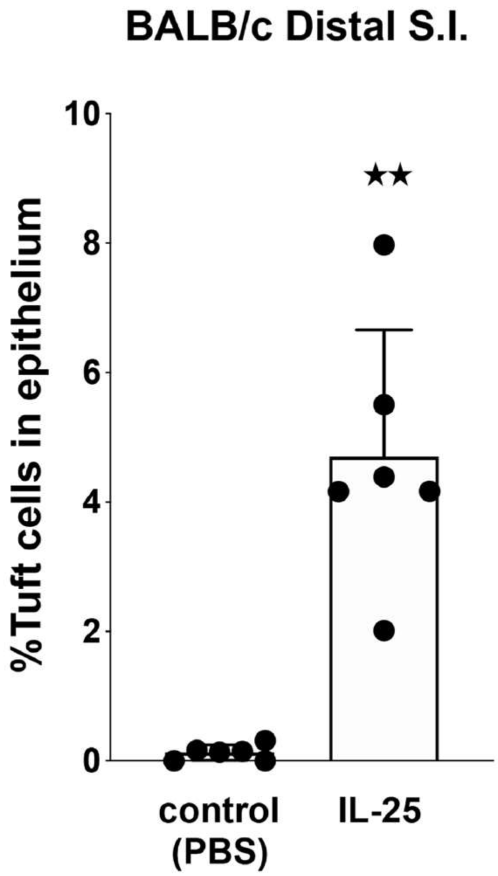

Figure S1. BALB/c mice expand distal SI tuft cells in response to IL-25. Tuft cell abundance determined by immunofluorescence after 1 week of IL-25 injection. Each symbol represents an individual mouse, and data are pooled from two independent experiments. Data are plotted as means with SEM. Two stars, $P < 0.01$. Mann-Whitney test.

Fig. S2

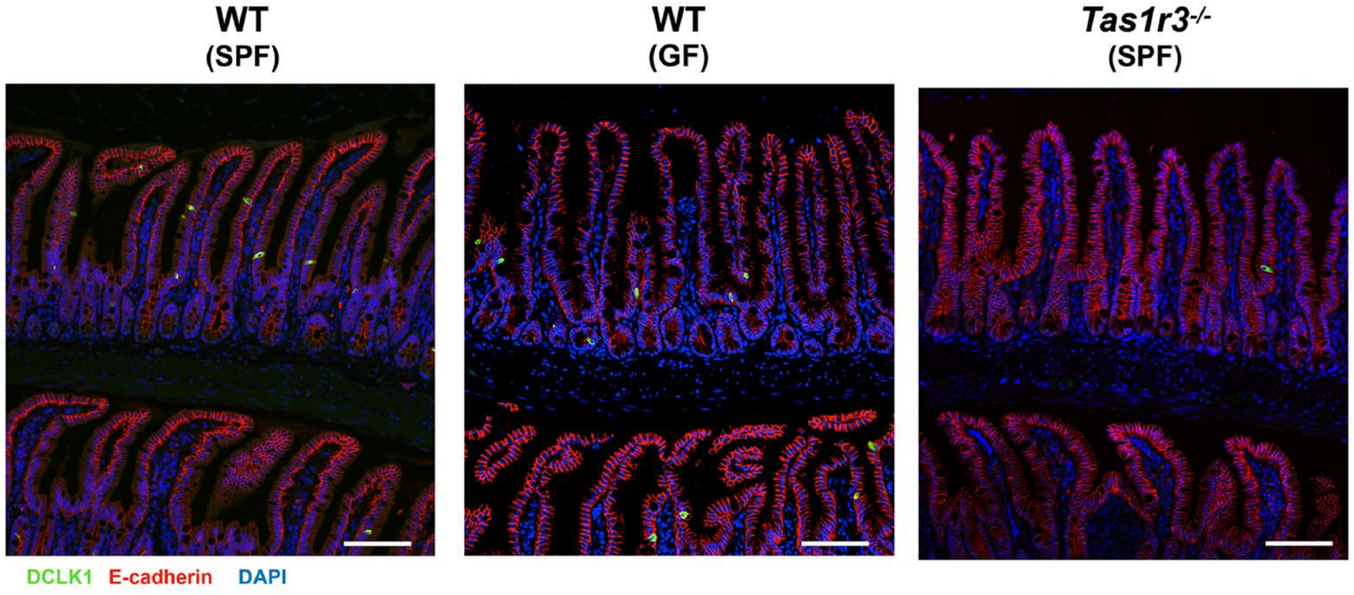

Figure S2. Distal SI tuft cells in C57BL6/J WT specific-pathogen-free (SPF), WT germ free (GF) and *Tas1r3*$^{-/-}$ (SPF) mice. Scale bars, 100 µm.

Fig. S3

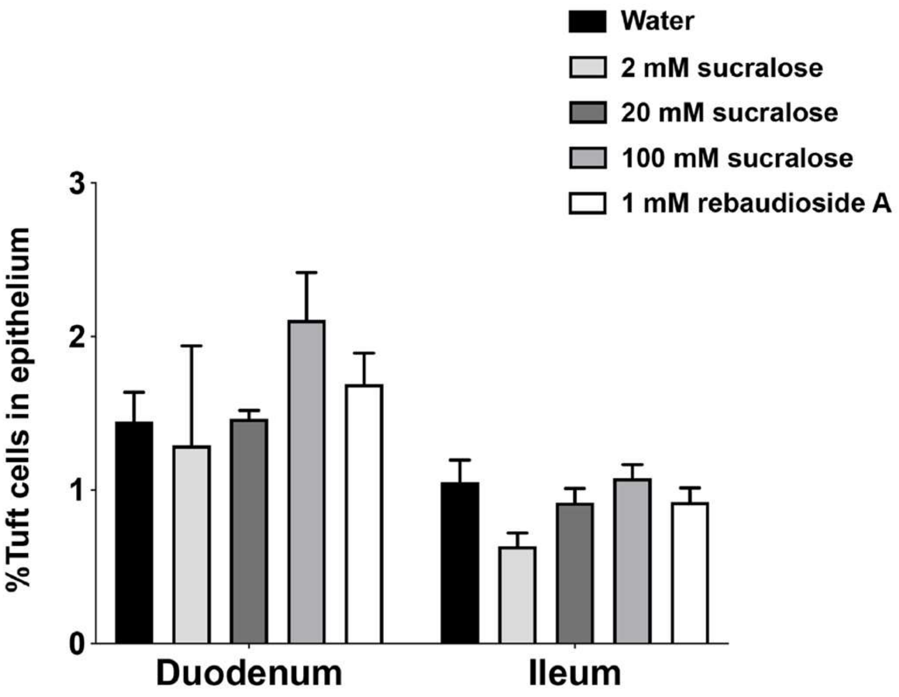

Figure S3. Taste pathway agonists do not expand intestinal tuft cells. Tuft cell abundance determined by flow cytometry after WT (*Gfi1b*$^{eGFP/+}$) C57BL/6J mice were fed sucralose or rebaudioside A in the drinking water at the indicated concentrations for 8 days. Data are representative of 2 to 8 biological replicates per group and plotted as means with SEM.
